# Obesity and Endocrine Dysfunction Programmed by Maternal Smoking in Pregnancy and Lactation

**DOI:** 10.3389/fphys.2012.00437

**Published:** 2012-11-19

**Authors:** Patricia Cristina Lisboa, Elaine de Oliveira, Egberto Gaspar de Moura

**Affiliations:** ^1^Laboratory of Endocrine Physiology, Department of Physiological Sciences, Roberto Alcantara Gomes Biology Institute, State University of Rio de JaneiroRio de Janeiro, Brazil

**Keywords:** gestation, lactation, programming, tobacco, nicotine, obesity, hormones

## Abstract

Obesity is a global epidemic, and maternal smoking has been shown to be associated with the development of childhood obesity. Overall, approximately 40% of children worldwide are exposed to tobacco smoke at home. It is well known that environmental changes within a critical window of development, such as gestation or lactation, can initiate permanent alterations in metabolism that lead to diseases in adulthood, a phenomenon called programming. It is known that programming is based on epigenetic alterations (changes in DNA methylation, histone acetylation, or small interfering RNA expression) that change the expression pattern of several genes. However, little is known concerning the mechanisms by which smoke exposure in neonatal life programs the adipose tissue and endocrine function. Here, we review several epidemiological and experimental studies that confirm the association between maternal nicotine or tobacco exposure during gestation or lactation and the development of obesity and endocrine dysfunction. For example, a positive correlation was demonstrated in rodents between increased serum leptin in the neonatal period and exposure of the mothers to nicotine during lactation, and the further development of leptin and insulin resistance, and thyroid and adrenal dysfunction, in adulthood in the same offspring. Thus, a smoke-free environment during the lactation period is essential to improving health outcomes in adulthood and reducing the risk for future diseases. An understanding of the pathophysiological mechanisms underlying the effects of smoking on programming can provide new insights into therapeutic strategies for obesity.

## Worldwide Epidemics of Overweight and Obesity

Obesity among adults, children, and adolescents has become an urgent problem and reached significant levels in recent years. The World Health Organization (WHO) (2011a) declared obesity a public health problem in 1997. Approximately 65% of the world’s population lives in countries where overweight and obesity kills more people than malnutrition. In 2010, the latest analysis of the IASO/IOTF (International Obesity Taskforce, 2010) estimates that approximately one billion adults are currently overweight and 475 million are obese. Globally, the IASO/IOTF estimates that up to 150 million school-aged children are overweight and 50 million are obese. Nearly 43 million children under 5 years old were overweight in 2010, particularly in urban settings (International Obesity Taskforce, 2010).

A higher body weight is associated with an increased risk of developing chronic diseases, including diabetes mellitus, cardiovascular disease, non-alcoholic fatty liver disease, dyslipidemia, and some of the most common cancers, alongside increased risk of disability (Walls et al., 2012). Although obesity is highly related to genetic factors, other factors may contribute to the high prevalence. A strong association has been shown between changes caused by epigenetic insults that occur *in utero* or early childhood (especially during the lactation period) that impact the regulation of metabolic pathways related to adipogenesis and complications, including diabetes, hypertension, and dyslipidemia (Monteiro et al., 2004; Doak et al., 2005; Ozanne et al., 2011).

## Worldwide Tobacco Consumption

One of the most common pollutants is exposure to cigarette smoke. In 2011, the WHO reported on the Global Tobacco Epidemic, warning about the dangers of tobacco and the impact of smoking cessation interventions (World Health Organization (WHO), 2011b). This same study predicted that if current trends continue, by 2030, smoking will kill one in six people, and the number of female smokers will significantly increase. In addition, this study showed that tobacco is a risk factor for six of the eight leading causes of death worldwide, including cardiac ischemia, pulmonary obstruction, and cerebrovascular diseases, and that most of the second-hand exposures were at home (54 vs. 37% at workplaces).

Globally, between 80,000 and 100,000 children start smoking every day [World Health Organization (WHO), 2009]. Given these data, it is impossible not to consider the effects of cigarette smoke on children. Approximately 40% of children worldwide are exposed to cigarette smoke at home, and 43% have at least one parent who smokes (World Health Organization (WHO), 2009). This early exposure to constituents of cigarette smoke makes children susceptible to respiratory infections, ear infections, growth retardation, and an increased frequency of hospitalizations due to infections (Ladomenou et al., 2009; Yilmaz et al., 2009a). Moreover, these children have decreased serum antioxidant vitamins (A, C, and E), which can contribute to an oxy-cellular redox imbalance and, consequently, increased lipid peroxidation and protein and DNA damage by free radicals present in cigarette smoke (Yilmaz et al., 2009b). Cigarette smoke exposure during pregnancy is related to reduced birth weight, a higher prevalence of sudden infant death syndrome, and impaired child growth, including decreased body mass, length, and head circumference at 3 months of age (Hegaard et al., 2006; Fenercioglu et al., 2009). When cigarette smoke exposure occurs during lactation, changes in milk composition, such as lower total lipid content during the first 6 months of lactation, are observed. Maternal tobacco use also changes cytokine concentrations in human milk; for example, the IL-1 concentration in the colostrum is lower in mothers who smoke compared to non-smoking mothers (Zanardo et al., 2005).

Cigarettes contain numerous toxic compounds, the most important of which are polycyclic aromatic hydrocarbons, nicotine, thiocyanate, and carbon monoxide (Passey et al., 1971). Nicotine, the main addictive compound of tobacco smoke, is an alkaloid produced in the roots of the tobacco plant and has a half-life of 2 h in humans. When nicotine is inhaled, it is quickly transported through the bloodstream across the blood brain barrier and cellular membranes (Rosemberg, 2002). Nicotine binds to nicotinic receptors present in neuromuscular junctions, synapses of the central nervous system and peripheral ganglion (Taylor, 1996).

In adulthood, smoking has controversial effects on body weight and adiposity (Chiolero et al., 2008). It has been shown that smoking decreases body weight, and the smoker becomes heavier after smoke cessation (Molarius et al., 1997). However, some studies have shown the contrary; heavy and chronic smokers had an increased BMI (body mass index), visceral adiposity, and insulin resistance (Ronnemaa et al., 1996; John et al., 2005). The increased visceral adiposity may be explained by the observations that cigarette smoke induces corticosterone secretion (Friedman et al., 1987), decreases testosterone in males (Meikle et al., 1988), and increases androgen in females (Khaw et al., 1988), which are conditions associated with higher visceral fat.

During pregnancy, nicotine crosses the placenta and affects fetal development (DiFranza and Lew, 1995). During lactation, nicotine is transferred through the maternal milk, and the main metabolite, cotinine, can be found in the urine of neonates (Laurberg et al., 2004).

## Programming

Stimuli or events that occur during critical periods of life, such as gestation and lactation, can shape future health and disease (Barker, 2003). This phenomenon was first defined as metabolic programming and is now known as developmental plasticity due to its probabilistic rather than deterministic nature (Gluckman and Hanson, 2007; Gluckman et al., 2010). Programming appears to play an important role in the development of obesity, which has raised concerns throughout the international scientific community and led to the creation of a society for the study of health maintenance and origin of diseases during development (DOHaD – Developmental Origins of Health and Disease). Obesity is considered a multifactorial disease, where events, which occur in a critical window of life can act as triggers or adjuncts, making the subject more susceptible to developing metabolic diseases (Walls et al., 2012). Among these events, maternal exposure to different factors, such as nutrients, hormones, and environmental pollutants, plays an important role in obesity development of the offspring (Moura and Passos, 2005; de Moura et al., 2008). In fact, some environmental and dietary chemicals that can mimic or interfere with the normal action of several hormones are termed “endocrine disruptors.” Studies predict the existence of chemical “obesogens,” molecules that inappropriately regulate lipid metabolism and adipogenesis to promote obesity (Grun and Blumberg, 2006; Tabb and Blumberg, 2006).

**Figure 1:**
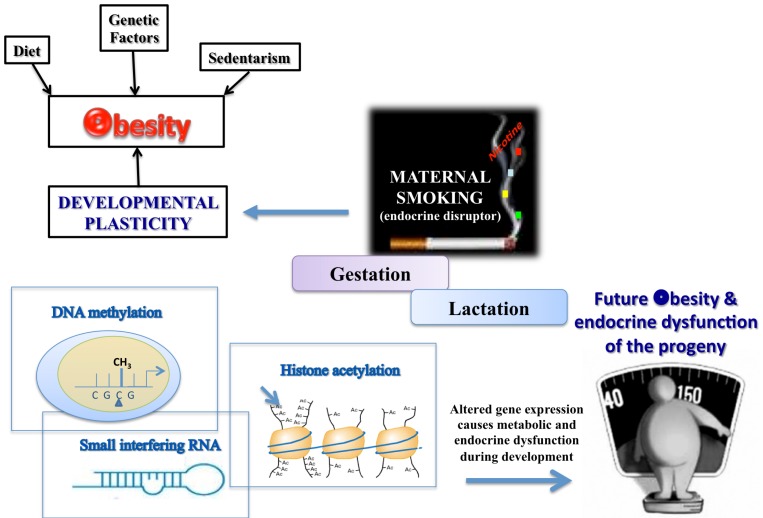
**TOBACCO – one of the environmental disruptors in obesity and endocrine dysfunction development of the offspring**.

As schematized in Figure 1, different priming factors could act through different epigenetic mechanisms. It appears that the obesogens could activate the processes of DNA methylation and histone acetylation or increase the levels of some small interference RNAs (siRNA), which can suppress genes related to normal adipogenesis, thereby affecting the developmental plasticity of the adipose tissue depending on the cumulative nutritional status during life (Gluckman et al., 2007; Guilloteau et al., 2009). Epidemiological studies of adults exposed *in utero* to calorie restriction during the Dutch Hunger in World War II were the first in which DNA hypomethylation at the imprinted *IGF2* region in mononuclear cells was demonstrated (Heijmans et al., 2008). Those undernourished babies developed into overweight adults who had a higher cardiovascular risk, confirming the hypothesis that the early life environment can cause epigenetic changes that persist throughout life and provide a mechanistic base for the DOHaD theory. In addition, Suter et al. (2010, 2011a) showed that *in utero*, tobacco exposure epigenetically modifies placental CYP1A1 expression through a decrease in DNA methylation, and this gene imprinting could explain the lower birth weight of babies of smoking mothers. More recently, the same group (Suter et al., 2011b) showed that a high fat maternal diet in non-human primates during gestation increases histone acetylation at the liver Npas2 gene promoter, which is related to circadian rhythms of lipid and glucose metabolism. Studies have suggested that perinatal tobacco exposure, likely due to the effects of nicotine, induces higher histone acetylation through decreases in the expression and activity of the sirtuins, mainly sirtuin 1, leading to a change in the oxidative stress and inflammatory profile in the offspring (Yang et al., 2007; Volkow, 2011). More information on epigenetic issues has been presented in several recent reviews (Waterland et al., 2008; Hussain, 2012).

## Tobacco and Programming

### Epidemiological and clinical data

Approximately 250 billion women smoke daily worldwide. Despite the well known harmful effects, many women continue to smoke throughout pregnancy. In the USA, 12% of women continue to smoke during pregnancy, resulting in the birth of at least 400,000 smoke-exposed children per year [Centers for Disease Control and Prevention (CDC), 2012; Martin et al., 2003]. Smoking has been associated with changes in gestation time, miscarriages, and shorter breastfeeding duration (Mello et al., 2001; Einarson and Riordan, 2009). During pregnancy, tobacco smoking is considered a risk factor for perinatal mortality, low birth weight, and neurological abnormalities (DiFranza and Lew, 1995). Nicotine acts as a potent vasoconstrictor, reducing the oxygen supply to the fetus and affecting development. During lactation, nicotine is transferred through the breast milk (Luck and Nau, 1987; Dahlström et al., 2004) and can act directly on the newborn’s metabolism. In fact, cotinine can be detected in the urine of neonates from smoking mothers. Suckling from a smoking mother has been shown to cause tachycardia in an infant, suggesting an adrenergic effect and demonstrating that nicotine causes important changes in breastfeeding infants (Laurberg et al., 2004).

Several reports have demonstrated an association between maternal smoking during pregnancy and the development of obesity and hypertension in childhood and adolescence (Von Kries et al., 2002; Wideroe et al., 2003; Goldani et al., 2007). However, it is not clear how different effects of maternal smoking can be synergistic or additive in the development of obesity in the offspring of smoking mothers. In a Chinese population, some parameters of metabolic syndrome, such as hypertriglyceridemia, central obesity, a decreased HDL-c and an increased level of fasting serum insulin in the adult offspring, have been associated with maternal cigarette smoke exposure (Xie et al., 2010). Metabolic changes in children exposed to cigarette smoke are related to the risk of developing atherosclerosis and diabetes. Epidemiological studies have shown decreased serum HDL-c in children exposed to cigarette smoke and increased markers of cardiovascular risk, including leptin, C-reactive protein, and IL-6, and decreased adiponectin concentrations (Nagel et al., 2009). Smoke exposure is also associated with the presence of autoantibodies to pancreatic islet cells, which would be the first step in the development of type 1 diabetes (Skrodenienė et al., 2008).

Additionally, tobacco smoke affects hormonal secretion, an effect that can program late endocrine dysfunctions. After the consumption of a few cigarettes, increases in ADH, GH, glucocorticoids, epinephrine, and leptin can be observed (Yeh and Barbieri, 1989; Grassi et al., 1994; Walker et al., 1999; Reseland et al., 2005). Even when non-smokers inhale tobacco smoke, it can act on different endocrine-metabolic parameters. In studies in which volunteers were subjected to environmental tobacco smoke, similar to a profile found in a bar/restaurant, increases of resting energy expenditure and serum thyroid hormones were observed with only 1 h of exposure (Metsios et al., 2007). In a similar study, the same increase in thyroid hormones was observed, along with increased production of the pro-inflammatory cytokine IL-1β and increased systolic blood pressure (Flouris et al., 2008). These hormonal changes should be further investigated because children of smoker mothers may have a higher risk of developing endocrine diseases later in life.

### Experimental data

For a better understanding of the role of tobacco smoke in the pathophysiology of obesity, researchers have used experimental models that allow the study of each tobacco component that may be involved in the development of obesity and, thus, provide a mechanistic basis prevention and treatment.

Experimental data have confirmed the epidemiological data associating maternal smoking during pregnancy with the development of obesity (Gao et al., 2005; Holloway et al., 2005; Somm et al., 2008). Williams and Kanagasabai (1984) showed that fetal nicotine exposure in rats increases body fat in the fetus on the 20th day of gestation (birth on the 21st day), suggesting that fetal nicotine exposure results in increased adiposity in the offspring. Newman et al. (1999) observed that rats exposed to nicotine *in utero* are heavier at 9 weeks of age compared with controls. In rodents, nicotine exposure from gestation to the 10th day of lactation increased body weight in offspring at 35 days of age. In male offspring, this effect was transient, but in female offspring, the higher body weight persisted until 90 days of age (Chen and Kelly, 2005). In addition, rats whose mothers were treated with nicotine for 14 days before mating and during pregnancy until weaning were heavier at 70 days of age compared with controls and had higher body weight and visceral adipose tissue at 180 days of age (Gao et al., 2005). In addition to obesity, adult animals whose mothers were exposed to nicotine during both gestation and lactation also display insulin resistance, glucose intolerance, cold intolerance, a reduction in spontaneous physical activity, and higher risk of developing cardiovascular diseases (Bruin et al., 2007; Somm et al., 2008, 2009).

It is interesting to note that most studies have investigated the effects of nicotine in rodents during gestation, which is equivalent in terms of neural development to the first two trimesters of gestation in humans. Considering neural development, lactation in rats is thought to be equivalent to the third trimester of human gestation (Quinn, 2005; Clancy et al., 2007). For eutherian mammals, the function of lactation is to provide the primary source of nutrition and immune protection to the offspring after birth (Golding et al., 1997). Because this period is critical to cognitive and neurological development, adverse environmental changes can predispose newborns to the development of some diseases in adulthood (Mott et al., 1991; Symonds, 2007). Nevertheless, despite experimental data of smoking/nicotine programming during pregnancy or gestation and lactation, to the best of our knowledge, there are few studies that have evaluated the effects of nicotine or tobacco exposure exclusively during the early postnatal period. This may be of particular relevance because there is a high rate of smoking relapse among women who quit smoking during pregnancy (McBride and Pirie, 1990). In fact, our group suggested that the postnatal period is critical for nicotine programming of body weight and fat distribution. Despite the differences in the neural development of rats and humans, studies that focus exclusively on the lactation period are important in evaluating the possible programming effects of neonatal exposure to cigarette smoke.

#### Hormonal changes associated with nicotine/tobacco exposure during lactation

Our group has shown that maternal nicotine exposure (6 mg/kg/day) from day 2 to 16 of lactation causes neonatal hyperleptinemia and primary hypothyroidism and programs for overweight, higher visceral adiposity, adipocyte hypertrophy, hyperleptinemia, leptin and insulin resistance, and secondary hypothyroidism in adult rat offspring (Oliveira et al., 2009; de Oliveira et al., 2010).

Increased body fat is associated with higher levels of leptin (Friedman and Halaas, 1998); however, the association between tobacco/nicotine and leptin levels is still controversial because both hyperleptinemia (Hodge et al., 1997; Eliasson and Smith, 1999; Nicklas et al., 1999) and hypoleptinemia (Wei et al., 1997; Donahue et al., 1999) have been found in tobacco smokers. The significance of higher serum leptin levels and the effect on leptin action are unclear because in most cases, it is associated with leptin resistance. Nevertheless, leptin resistance can be tissue-specific. It has been shown (Rahmouni et al., 2008) that in some knockout mice models of Bardet–Biedl obesity syndrome, leptin resistance has anorexigenic effects; however, leptin preserves its effects on renal sympathetic nerve activity. Our group also demonstrated that even in the sympathetic nervous system (SNS), a peripheral adrenal medulla leptin resistance is present, while the central leptin action upon the SNS is preserved (Trevenzoli et al., 2010).

Some leptin effects on energy balance may be mediated by the hypothalamus-pituitary-thyroid axis and peripheral metabolism of thyroid hormones (Ahima et al., 1996; Légradi et al., 1997; Friedman and Halaas, 1998; Seoane et al., 2000; Nowak et al., 2002; Ortiga-Carvalho et al., 2002; Lisboa et al., 2003). In fact, disturbances of thyroid function are associated with marked changes in both energy expenditure and body weight, with leptin and thyroid hormones appearing to play mutually important roles. It is well known that tobacco can affect the thyroid gland (Christensen et al., 1984; Ericsson and Lindgrade, 1991; Fisher et al., 1997; Utiger, 1998), and although there are few studies concerning the effect of tobacco compounds on thyroid function, thiocyanate, not nicotine, is frequently related to hypothyroidism and goiter (Muller et al., 1995; Dai et al., 1996; Fukata et al., 1996; Eskandari et al., 1997). Maternal smoking influences the thyroid function of the newborn differently, likely according to the levels of iodine-intake. In newborns of normal iodine-intake mothers, it appears that smoke is related to neonatal hyperthyroidism suppressing TSH (Meberg and Marstein, 1986), while in borderline iodine-intake mothers, smoke exposure is associated with goiter (Chanoine et al., 1991). According to Laurberg et al. (2004), smoking mothers have lower iodide content in breast milk, and their offspring have lower urinary iodide. This study suggests that nicotine decreases maternal milk iodide transfer.

**Figure 2:**
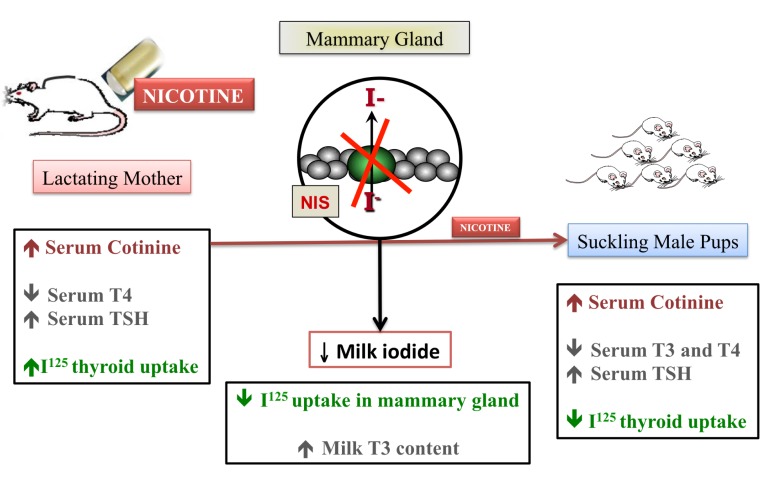
**The anti-thyroid effect of nicotine during lactation**.

As we previously described thyroid hypofunction in rat pups after maternal exposure to nicotine, we decided to evaluate the transfer of iodine through milk in this model (de Oliveira et al., 2011). As schematized in Figure 2, during nicotine exposure, mothers showed decreased serum T4 and mammary radioiodine uptake (RAIU) and increased serum TSH and thyroid RAIU, followed by higher T3 content in milk. In addition to decreased T3 and T4 and increased TSH levels, pups from nicotine mothers showed lower thyroid RAIU. Interestingly, at weaning after nicotine withdrawal, the pups recovered thyroid function, likely due to the increased lactational transfer of T3. Therefore, we demonstrated that the thyroid function of both mothers and pups was affected by early nicotine exposure. The likely mechanism of the pups’ primary hypothyroidism is decreased leptin signaling in the thyroid. However, long-term thyroid hypofunction is related to central and peripheral leptin resistance in adulthood (Santos-Silva et al., 2010).

In another study (Pinheiro et al., 2011), we investigated the association of leptin and adrenal hormones in adult male offspring whose mothers were treated with nicotine during lactation. These progeny presented with higher production of adrenal catecholamine but lower catecholamine secretion. They also displayed hypothalamic-pituitary-adrenal axis hyperfunction, characterized by an increase in both CRH and ACTH immunostaining in the hypothalamus and pituitary and higher serum corticosterone levels. Because obesity depends on SNS and hypothalamic-pituitary-adrenal function, these findings could be associated with a higher probability of developing obesity due to lower catecholamine secretion (reducing the lipolytic effects of SNS) and higher corticosterone action (increasing visceral adipogenesis).

Thus, our studies identified lactation as a critical period for nicotine programming for obesity, with both thyroid and adrenal dysfunctions being possible contributing factors. In addition, through the effects of nicotine, perinatal maternal smoking can be responsible for the future development of some components of the metabolic syndrome in offspring.

Table 1 presents some findings concerning a sex dimorphism in a model of maternal nicotine exposure during lactation. Adrenal function in adult offspring was programmed only in male offspring, while female offspring showed only changes in leptin content in muscle and adipocytes (Pinheiro et al., 2011). Thus, males may be more susceptible to maternal smoke-induced obesity than females. It is possible that the resistance to the programming found in female rats is an evolutionary advantage because the environmental changes are not fully transmitted to the progeny. However, there is lack of epidemiological studies to support this hypothesis.

**Table 1:** **Comparison between some parameters of adult (180 days of age) male and female rat progeny whose mothers were nicotine-treated only during lactation period**.

180 days of age	Male offspring	Female offspring
**BIOMETRIC PARAMETERS**		
Visceral adipose tissue mass	↑	Unchanged
Total body fat mass	↑	↓
Subcutaneous fat mass	Unchanged	↓
Total body protein	↑	↓
**SERUM HORMONES**		
Leptin	↑	Unchanged
Corticosterone	↑	Unchanged
**ADRENAL PARAMETERS**		
Gland mass	Unchanged	Unchanged
Catecholamine content	↑	Unchanged
Tyrosine hydroxylase	↑	Unchanged
**TISSUE PROTEIN CONTENT**		
Leptin in visceral adipocyte	↑	Unchanged
Leptin in subcutaneous adipocyte	Unchanged	↓
Leptin in soleus muscle	Unchanged	↓
Adrenergic β3 receptor in visceral adipocyte	↑	↓

A cigarette contains more than 4,500 compounds in addition to nicotine. Therefore, it is interesting to evaluate the effects of total cigarette exposure during lactation. To evaluate this possible combined programming effect of cigarette compounds, we more recently developed a model of maternal cigarette smoking exposure during lactation to investigate some nutritional, biochemical, and hormonal parameters in dams and pups. In this experiment, lactating rats were submitted to a smoking machine 4 times/day/h for each exposure (1.7 mg nicotine/cigarette) during the lactation period without pups to attempt to reproduce the human conditions in which the lactating mother only smokes far from her baby. Different from the pups whose mothers were exposed solely to nicotine, the pups from smoke-exposed mothers did not exhibit neonatal thyroid dysfunction and had lower body adiposity. In addition, they had lower adrenal catecholamine content and lower serum corticosterone levels at weaning (Santos-Silva et al., 2011). The only programming effect noted in the offspring was decreased catecholamine production in the adrenal gland at adulthood, a dysfunction that can lead to long-lasting changes in the stress response. However, these adult offspring surprisingly had no changes in body adiposity or serum hormone profile (Santos-Silva et al., 2012). Other cigarette components may inhibit the obesogenic effect of nicotine, or more likely, the time period of exposure (4 h/day) was not adequate to transfer enough nicotine to the pups. Another point to consider is that the newborn may be exposed to cigarette smoke in the environment because relatives other than the mother, such the father, can also smoke. Thus, we are studying another model in which the pups stay with the mother during the full period of cigarette smoke exposure in the smoke chamber.

To clearly elucidate the effect of tobacco programming, Tables 2 and 3 depict the major changes found into two models of smoke exposure during lactation in rodents, namely, maternal nicotine exposure (Oliveira et al., 2010) and cigarette smoke exposure (Santos-Silva et al., 2011). Our comparison of these two experimental models suggest that some changes result specifically from nicotine exposure, such as thyroid and adrenal dysfunction, serum HDL-c and leptinemia. Surprisingly, some of the nicotine effects were blunted when the animals were exposed to the cigarette smoke, such as thyroid dysfunction and hyperleptinemia. In addition, components of tobacco other than nicotine may be responsible for the alterations in triglycerides and LDL-c.

**Table 2:** **Comparison between milk composition in the models of maternal nicotine and tobacco smoke-exposed during lactation**.

	Nicotine	Tobacco
Cotinine	↑	↑
Calories	↑	↑
Lactose	↑	↑
Protein	Unchanged	Unchanged
Cholesterol	Unchanged	Unchanged
Triglycerides	Unchanged	↑

**Table 3:** **Comparison between metabolic and endocrine changes in the models of maternal nicotine and tobacco smoke-exposed during lactation**.

		Mother	Male pups
Serum cotinine	Tobacco	↑	↑
	Nicotine	↑	↑
Serum cholesterol	Tobacco	Unchanged	Unchanged
	Nicotine	Unchanged	Unchanged
Serum HDL-c	Tobacco	↑	↑
	Nicotine	↑	↑
Serum LDL-c	Tobacco	Unchanged	↓
	Nicotine	Unchanged	Unchanged
Serum VLDL-c	Tobacco	↑	Unchanged
	Nicotine	Unchanged	Unchanged
Serum triglycerides	Tobacco	↑	↑
	Nicotine	Unchanged	Unchanged
Serum leptin	Tobacco	Unchanged	Unchanged
	Nicotine	↑	↑
Serum triiodothyronine	Tobacco	Unchanged	Unchanged
	Nicotine	Unchanged	↑
Serum thyroxine	Tobacco	Unchanged	Unchanged
	Nicotine	↓	↓
Serum corticosterone	Tobacco	Unchanged	↑
	Nicotine	Unchanged	↑
Adrenal catecholamine content	Tobacco	Unchanged	↑
	Nicotine	Unchanged	↑

Our experimental data and data from the literature (experimental, clinical, and epidemiological) show that maternal smoking, mainly through nicotine/tobacco smoke exposure in early life, can significantly influence neonate development, with an emphasis on obesity.

## Concluding Remarks – Perspectives

Smoking during pregnancy and lactation remains a major scientific and public health concern and are associated with numerous short- and long-term adverse effects. We can learn from the developmental programming induced by maternal smoking, and its future impact on endocrine dysfunction and obesity that in addition to its clear toxic effects, tobacco smoke has long-term effects contributing to the obesity epidemic.

We do not yet know all of the mechanisms by which this early life exposure leads to the observed changes in adipogenesis or hormonal changes, including epigenetic alterations. Unanswered questions remain concerning the role of hormonal or transcript factors, such as PPARgamma, on the adipogenesis in this condition and how the different factors that are changed during the neonatal period combine to increase the probability of these reported developmental changes.

The possibility that tobacco compounds can make children who are exposed to cigarette smoke during the gestation and/or lactation periods, through blood cord, milk, or environment, more susceptible to obesity and other metabolic and endocrine disorders in adulthood warrants clinical, epidemiological, prospective, and experimental studies that support a more assertive health policy by governments toward education about the risk of cigarette smoke, at least in vulnerable periods of life, such gestation, lactation, and infancy.

## Conflict of Interest Statement

The authors declare that the research was conducted in the absence of any commercial or financial relationships that could be construed as a potential conflict of interest.
